# Gut microbiome-derived indoles differentially suppress type III secretion system-associated virulence in *Escherichia coli* and *Salmonella enterica*

**DOI:** 10.3389/fmicb.2026.1902638

**Published:** 2026-08-13

**Authors:** Michal Shvarthburg, David Taussig, Neta Even-Tov, Yariv Wine, Neta Sal-Man

**Affiliations:** 1 The Shraga Segal Department of Microbiology, Immunology, and Genetics, Faculty of Health Sciences, Ben-Gurion University of the Negev, Be'er-Sheva, Israel; 2 The Shmunis School of Biomedicine and Cancer Research, The George S. Wise Faculty of Life Science, Tel Aviv University, Tel Aviv-Yafo, Israel

**Keywords:** bacterial virulence, EPEC, indole derivatives, postbiotics, type 3 secretion system

## Abstract

Enteric pathogens such as enteropathogenic *Escherichia coli* (EPEC), enterohemorrhagic *E. coli* (EHEC), and *Salmonella enterica* utilize a conserved type III secretion system (T3SS) to manipulate host cell processes and promote infection. Metabolites present in the intestinal lumen can directly influence the activity of these systems. Indole, which is produced from dietary tryptophan, has been shown to suppress the T3SS function of enteric pathogens. Here, we investigated the anti-virulence potential of seven gut microbiome-derived indole derivatives and found that their ability to suppress T3SS-dependent virulence is highly structure-dependent. Whereas indole exhibited moderate inhibition, indole-3-carboxaldehyde (I3A) and 3-methylindole (3-MI) emerged as more potent suppressors. In practice, these metabolites downregulated transcription of key T3SS genes, leading to reduced secretion of T3SS translocators, impaired effector delivery into host cells, and a marked reduction in actin pedestal formation, a hallmark of EPEC infection. Notably, this inhibitory effect extended to EHEC and *Salmonella*, suggesting that these metabolites target conserved virulence regulatory pathways. Overall, our findings demonstrate that microbiome-derived indole metabolites can effectively attenuate pathogen virulence. By targeting virulence rather than bacterial viability, 3-MI and I3A emerge as promising postbiotic-like therapeutic leads that can mitigate infection while limiting selective pressure for resistance.

## Introduction

Enteric bacterial pathogens such as enteropathogenic *Escherichia coli* (EPEC) and enterohemorrhagic *E. coli* (EHEC) are major causes of diarrheal diseases globally and impose substantial health and economic burdens (Kaper et al., 2004; Khalil et al., 2018). These pathogens colonize the intestinal epithelium using the type III secretion system (T3SS), a conserved syringe-like nanomachine that delivers effector proteins directly into host cells (Cornelis, 2006; Galan and Wolf-Watz, 2006). These effectors rewire host signaling pathways that regulate cytoskeletal organization, inflammatory responses, and epithelial barrier integrity, thereby facilitating bacterial attachment and host cell infection while suppressing host immune defenses (Van Avondt et al., 2015).

In EPEC and EHEC infections, T3SS activity drives the formation of attaching-and-effacing (A/E) lesions on intestinal epithelial cells. These are formed when T3SS-delivered effectors stimulate localized actin polymerization beneath adherent bacteria, generating actin-rich pedestals that anchor the pathogen to the host cell surface (Battle et al., 2014; Campellone and Leong, 2003). These cytoskeletal rearrangements are essential for intimate bacterial attachment and contribute directly to successful colonization of the intestinal epithelium. Accordingly, disruption of T3SS function significantly reduces the ability of these bacteria to cause illness (Kumar et al., 2019; Lai et al., 2013).

The T3SS is encoded by the locus of enterocyte effacement (LEE), a pathogenicity island that harbors genes of structural components of the T3SS apparatus, several regulatory proteins, and multiple effector proteins. The LEE is regulated by Ler, its master transcriptional regulator, which integrates multiple regulatory inputs to control the timing and level of virulence gene expression. Upon activation, the T3SS secretes three translocator proteins - EspA, EspB, and EspD - that form key structural components of the secretion apparatus. EspA polymerizes into a long filamentous structure, while EspB and EspD hetero-oligomerize to assemble a pore complex in the host cell membrane. Following the assembly of the apparatus, effectors are delivered into the host cell. The first effector translocated is Tir (translocated intimin receptor), which becomes inserted into the host membrane and binds the bacterial outer membrane protein Intimin. This interaction mediates intimate attachment of the bacterium and triggers the formation of actin-rich pedestal structures (Ide et al., 2003; Leverton and Kaper, 2005; Mitrovic and Sal-Man, 2022; Slater et al., 2018).

The expression of T3SS components is activated exclusively within the intestinal niche in response to host-specific environmental signals, and not when EPEC and EHEC are present in the environment (Slater et al., 2018; Connolly et al., 2018; Sun et al., 2024; Zacharia et al., 2022). Within the host intestine, these pathogens encounter a complex metabolic landscape shaped by both host-derived compounds and metabolites produced by the resident microbiome. Accumulating evidence demonstrates that small molecules produced by commensal bacteria can profoundly influence pathogen behavior by regulating transcription of genes within the LEE, modulating T3SS activity dynamics, and ultimately altering virulence potential (Sun et al., 2024; Cottam et al., 2024; Pacheco and Sperandio, 2015; Platenkamp and Mellies, 2018).

One of the best-characterized metabolites known to affect T3SS activity is indole, a tryptophan-derived metabolite produced by many bacterial species via the enzyme tryptophanase (TnaA). This enzyme converts tryptophan into indole, pyruvate, and ammonia (Lee and Lee, 2010). Indole can accumulate to millimolar concentrations in the intestinal lumen (Hubbard et al., 2015; Ye et al., 2022) and has been shown to regulate diverse bacterial behaviors, including biofilm formation, motility, stress responses, and the expression of virulence-associated genes (Kumar et al., 2019; Lee and Lee, 2010; Tennoune et al., 2022). Previous work from our group and others has shown that indole suppresses virulence-associated phenotypes of enteric pathogens, including inhibition of T3SS activity and reduced effector translocation (Franco et al., 2025; Gorelik et al., 2022; Kumar et al., 2022). In addition, Bommarius et al. demonstrated that indole and a limited set of indole derivatives, primarily indole-3-carboxaldehyde, suppress virulence phenotypes, including pedestal formation and biofilm formation, in pathogenic *E. coli* (Bommarius et al., 2013). Building on these earlier findings, we systematically evaluated a broader panel of natural indole-derivatives to determine whether the observed anti-virulence activity is specific to particular indole derivatives.

**Figure 1:**
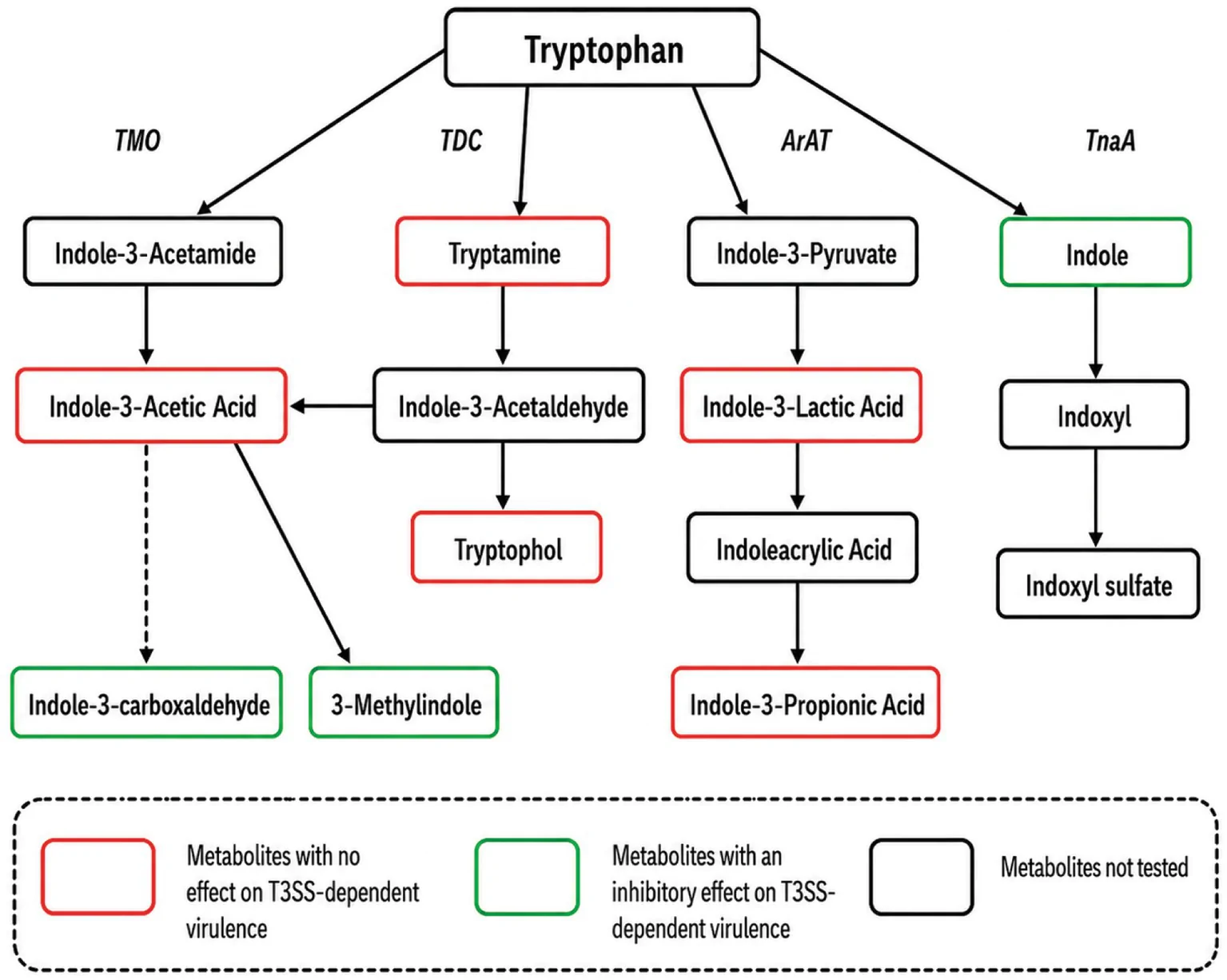
Bacterial tryptophan metabolism: pathways to indole derivatives. Tryptophan is metabolized by bacteria through multiple pathways, leading to the production of diverse indole-derived metabolites. These pathways are mediated by the enzymes Tryptophan 2-monooxygenase (TMO), Tryptophan decarboxylase (TDC), Aromatic amino acid aminotransferase (ArAT), and Tryptophanase (TnaA). The compounds examined in this study are color-coded: Metabolites that showed a T3SS-suppressive effect are marked in green, whereas those that did not are marked in red. Metabolites that were not used in this study are marked in black. Solid arrows indicate bacterial metabolic conversions supported by published experimental evidence, whereas the dashed arrow indicates a proposed metabolic conversion that is currently supported primarily by indirect genomic, metabolomic, and literature-based evidence rather than direct experimental validation in bacteria.

Indole belongs to a broad family of tryptophan-derived metabolites that share a conserved indole ring but differ in their side-chain substitutions (Figure 1) (Dodd et al., 2017; Honeyfield and Carlson, 1990; Li et al., 2021; Lu et al., 2021; Roager and Licht, 2018; Spaepen et al., 2007; Whitehead et al., 2008; Zhang et al., 2025). Many of these indole-derived metabolites are naturally present in the intestinal environment and can influence bacterial virulence pathways (Ye et al., 2022; Powell et al., 2020; Ranhotra, 2024). Notably, indole shares structural similarities with serotonin, a biologically active tryptophan metabolite whose regulatory constraints complicate its use in research and clinical settings (Costantini et al., 2020). Given that multiple indole derivatives are products of microbial tryptophan metabolism (Hyland et al., 2022), we hypothesized that a broader panel of these compounds could similarly modulate T3SS-mediated virulence. Accordingly, we examined a panel of seven indole derivatives (3-methylindole, indole-3-carboxaldehyde, 3-indoleacetic acid, indole-3-lactic acid, indole-3-propionic acid, tryptamine, and tryptophol) for their ability to influence T3SS activity and related pathogenic behavior.

## Materials and methods

### Bacterial strains

The wild-type (WT) EPEC O127: H6 strain E2348/69 [streptomycin-resistant] (Iguchi et al., 2009) and its isogenic Δ*escN* mutant strain (Gauthier et al., 2003), the WT EHEC O157: H7 strain EDL933 (Saile et al., 2016) and its corresponding Δ*escN* mutant (Clements et al., 2014), as well as WT *Salmonella enterica* serovar Typhimurium SL1344 [streptomycin-resistant] (Kujat Choy et al., 2004) and its corresponding Δ*invA* mutant [SB103] (Steele-Mortimer et al., 2002), were used to assess T3SS activity and host-cell infection (Table 1). The bacteria were grown in Luria-Bertani (LB) broth or high-glucose Dulbecco’s Modified Eagle’s Medium (DMEM, Biological Industries Ltd.), and supplemented with streptomycin when appropriate.

**Table 1:** Bacterial strains used in this study.

Strain	Description	Reference
WT EPEC	EPEC strain E2348/69, streptomycin resistant	Iguchi et al. (2009)
EPEC Δ*escN*	Nonpolar deletion of *escN*, streptomycin resistant	Gauthier et al. (2003)
WT EHEC	EHEC strain EDL933	Saile et al. (2016)
EHEC Δ*escN*	Nonpolar deletion of *escN*	Clements et al. (2014)
WT *Salmonella enterica* serovar Typhimurium	*Salmonella* strain SL1344, streptomycin resistant	Kujat Choy et al. (2004)
*Salmonella enterica* serovar Typhimurium Δ*invA*	*Salmonella* strain SB103, streptomycin resistant	Steele-Mortimer et al. (2002)

### Indole and indole derivatives

Indole (CAS No.120–72-9, Sigma-Aldrich), 3-methylindole (3-MI; CAS No. 83–34-1, Sigma-Aldrich), indole-3-carboxaldehyde (I3A; CAS No. 487–89-8, Sigma-Aldrich), 3-indoleacetic acid (3-IAA; CAS No. 87–51-4, Sigma-Aldrich), indole-3-lactic acid (I3LA; CAS No. 832–97-3, Sigma-Aldrich), indole-3-propionic acid (I3PA; CAS No.830–96-6, Sigma-Aldrich), tryptamine (CAS No. 61–54-1, Sigma-Aldrich), tryptophol (CAS No.526–55-6, Sigma-Aldrich), were dissolved in dimethyl sulfoxide (DMSO) to prepare 100 mM stock solutions. These stock solutions were then diluted to the indicated concentrations in the relevant media immediately before use. In all experiments, WT cultures not treated with indole derivatives received an equivalent volume of DMSO alone as a vehicle control.

### T3SS activity assay

The activity was assessed by measuring the secretion of T3SS substrates into the culture supernatant following induction, as previously described in Mitrovic and Sal-Man (2022), Gorelik et al. (2022), and Mitrovic et al. (2021). Briefly, overnight EPEC cultures grown in LB broth were diluted 1:100 into pre-warmed high-glucose DMEM supplemented with either DMSO alone (vehicle control) or indole derivatives dissolved in DMSO at the indicated concentrations (100–1,000 μM) and incubated statically for 6 h in a tissue culture incubator (5% CO₂, at 37 °C) to an optical density of ~0.7 at 600 nm (OD_600_). Cultures were then centrifuged at 20,000 × *g* for 5 min; the bacterial pellets were resuspended in SDS-PAGE sample buffer; and the supernatants were collected, normalized according to cultures’ OD_600_ values, and filtered through a 0.22 μm low-protein-binding filter (Millipore) to remove residual cells and debris. The secreted proteins found in the supernatant samples were precipitated by adding trichloroacetic acid (TCA) to a final concentration of 10% (v/v), followed by overnight incubation at 4 °C. Samples were then centrifuged at 18,000 × *g* for 30 min at 4 °C. The secreted proteins were resuspended in SDS-PAGE sample buffer, and residual TCA was neutralized using saturated Tris buffer. Samples were subsequently loaded on SDS-PAGE and analyzed by Coomassie Blue staining (InstantBlue, Abcam) or by western blotting with antibodies against specific T3SS components. T3SS activity in EHEC was assessed using a similar protocol, except that the bacteria were cultured for 8 h with twice the culture volume used for EPEC. For *Salmonella enterica* serovar Typhimurium, T3SS activity was evaluated by culturing bacteria in LB and incubating them at 37 °C with shaking for 3 h. Following incubation, culture supernatants were collected as described above, and secreted proteins were precipitated using TCA. The resulting protein pellets were washed with cold acetone, air-dried, and resuspended in SDS-PAGE sample buffer prior to SDS-PAGE analysis.

### Host cell infection and effector translocation assay

Experiments were performed as previously described by Baruch et al. (2011). Briefly, WT EPEC and Δ*escN* strains were primed for T3SS activation by growing them for 3 h under T3SS-inducing conditions (DMEM, in a 5% CO_2_ tissue culture incubator, statically) in the presence or the absence of indole derivatives (500–2,000 μM). Bacteria were then used to infect HeLa cells (8 × 10^5^ cells) at a multiplicity of infection (MOI) of 1:300 for 3 h, while maintaining the same concentrations of indole derivatives throughout the infection. At the end of the infection period, the cells were washed with cold PBS and lysed using RIPA buffer. The lysates were collected and centrifuged at 18,000 × *g* for 5 min to pellet intact cells. The cleared supernatants were then resuspended in SDS-PAGE sample buffer and analyzed by western blotting using a primary antibody against c-Jun N-terminal kinase (JNK), a cellular protein degraded by a translocated bacterial effector NleD. Blots were also probed with an anti-Actin antibody as a loading control. Lysates from uninfected cells and cells infected with the Δ*escN* strain served as negative controls.

### Lactate dehydrogenase (LDH) cytotoxicity assay

HeLa cells (5 × 10^4^ cells/well) were seeded in 96-well plates and allowed to adhere overnight. The culture medium was then replaced with fresh medium containing indole, 3-MI, or I3A at 500, 1,000, or 2,000 μM concentration for 4 h at 37 °C in a 5% CO₂ tissue culture incubator. A vehicle-control sample received DMSO alone at the corresponding final concentrations (0.5%, 1%, or 2%), and untreated cells were included as a negative control as well as cell-free background and maximum-LDH-release controls (according to the manufacturer’s instructions). At the end of incubation period, culture supernatants were collected and analyzed using the CytoTox 96® Non-Radioactive Cytotoxicity Assay (Promega) according to the manufacturer’s instructions. Absorbance was measured at 490 nm, and cytotoxicity was calculated relative to the maximum-LDH-release control. The assay was performed in three independent biological experiments, each including four technical replicates per condition.

### Western blotting

Proteins were separated by SDS-PAGE and transferred to nitrocellulose or PVDF membranes (pore size: 0.45 μm, Cytiva). Blots were blocked for 1 h in 5% (w/v) skim milk-PBST (0.1% Tween in phosphate-buffered saline [PBS]), incubated for 1 h with appropriate primary antibodies (diluted in 5% skim milk-PBST) at room temperature, washed, and incubated for 1 h with appropriate secondary antibodies (diluted in 5% skim milk-PBST) at room temperature. Chemiluminescent signals were detected using EZ-ECL reagents (Westar Antares, Cyanagen) and visualized using a chemiluminescence imaging system. The following antibodies were used: mouse anti-DnaK (Abcam, Inc.), diluted 1:5,000; rabbit anti-JNK (Abcam Inc.), diluted 1:1,000; mouse anti-Actin (MPBio), diluted 1:10,000. Antibodies directed against T3SS components were a generous gift from Prof. B. Brett Finlay (University of British Columbia, Canada) and Prof. Rebekeh DeVinney (University of Calgary, Canada) and included rat anti-EspB, diluted 1:500; mouse anti-Tir, diluted 2,000; rat anti-EspD, diluted 1:1,000; and rat anti-Intimin, diluted 1:2,000. Human anti-SipB was jointly developed together with the Wine group (Tel-Aviv University, Israel) and was used diluted 1:1,000. Horseradish peroxidase-conjugated (HRP)-goat anti-mouse/rat/rabbit/human (Abcam Inc.), diluted 1:10,000, were used as the secondary antibody for those analyses.

### Bacterial growth

To determine the effect of indole derivatives on bacterial fitness, overnight bacterial cultures (grown in LB broth at 37 °C with shaking) were diluted into fresh media as follows: EPEC and EHEC strains into phenol red–free DMEM and *Salmonella* strains in LB broth. The cultures were grown for 8 h at 37 °C in the absence or the presence of indole derivatives at concentrations of 1 mM or 2 mM, and OD_600_ was monitored at 1-h intervals. EPEC and *S.* Typhimurium strains were supplemented with streptomycin.

### Quantitative real-time PCR (qPCR)

To examine the effect of indole derivatives on the expression of T3SS-related genes, qPCR analysis was performed. Overnight cultures of EPEC and EHEC were diluted 1:100 into pre-warmed DMEM supplemented with indole derivatives (1 mM or 2 mM) and grown statically in a tissue culture incubator (with 5% CO_2_) for 3 h to the early exponential phase of growth. Total RNA was extracted from bacterial cultures using TRIzol and treated with DNaseI to remove residual genomic DNA. From each sample, 1 μg of total RNA was used for cDNA synthesis, which was carried out using the ProtoScript II First Strand cDNA Synthesis Kit (NEB) with a random primer mix. Before further processing, the resulting cDNA was confirmed to be free of genomic DNA contamination. qPCR was subsequently performed using gene-specific primers targeting the virulence-associated genes *ler*, *espB*, and *tir* (Table 2). The housekeeping gene *rpoA* was used as an internal control for normalization of gene expression levels (Gorelik et al., 2019; Hazen et al., 2015; Hazen et al., 2017). qPCR was performed using SYBR Green I Master Mix (Roche) with cDNA templates, and amplification was carried out on QuantStudio™ 1 Real-Time PCR System (Applied Biosystems, Thermo Fisher Scientific). The thermal cycling protocol consisted of an initial denaturation step at 95 °C for 10 min, followed by 40 cycles of denaturation at 95 °C for 15 s, annealing at 60 °C for 10 s, and extension at 72 °C for 10 s while monitoring fluorescence. The sequences of the primers used for the qPCR experiments are presented in Table 2. qPCR specificity was confirmed by melting curve analysis. Relative gene expression was calculated using the 2^-ΔΔ*CT*^ method, with normalization to the housekeeping gene rpoA, whose expression remained stable across the tested conditions, serving as the internal reference.

**Table 2:** Primers used for quantitative real-time PCR (qPCR) analysis.

Gene	Organism	Primer name	Primer sequence	Reference
*espB*	EPEC	EspB_qPCR_F EspB_qPCR_R	GGCTCTTTTGCTGCCATTAATAGC TCTGCTGCATCTGCAATACC	Gorelik et al. (2022)
*tir*	EPEC	Tir_qPCR_F Tir_qPCR_R	GGACCCTCTGCATTTCGTGTTG GTCCCCCGGTAAAAACAAATCTG	Gorelik et al. (2022)
*espB*	EHEC	EHEC_EspB_qPCR_F EHEC_EspB_qPCR_R	GCTTCGGAGAGTACGACCG CGGCCTGCTGAATCTGATAGC	Gorelik et al. (2022)
*tir*	EHEC	EHEC_Tir_qPCR_F EHEC_Tir_qPCR_R	CGCCTGTAAGGAATTCTATGGC GCCTGTTAAGAGTATCGAGCGG	Gorelik et al. (2022)
*ler*	EPEC/ EHEC	Ler_F_qPCR Ler_R_qPCR	GACTGCGAGAGCAGGAAGTT CCAGGTCTGCCCTTCTTCATTG	This study
*rpoA*	EPEC/ EHEC	rpoA_qPCR_F rpoA_qPCR_R	CAAGGTGACCCTTGAGCCTT TCCTGAACGCCTTCTTTGGTG	This study

### Actin pedestal formation assay

Fluorescence-based pedestal formation was assessed as previously described in Battle et al. (2014); Dhanda et al. (2021); Swistak et al. (2025); Vogt et al. (2023). Briefly, HeLa cells were seeded onto glass coverslips at a density of 3.5 × 10^4^ cells per well and allowed to grow for 2 days to reach optimal confluency. On the third day, EPEC strains were grown under T3SS-inducing conditions for 3 h in the absence or the presence of indole derivatives (1,000 μM for 3-MI and I3A and 2,000 μM for indole). Primed bacteria were normalized based on their OD_600_ values prior to infection. HeLa cells were infected for 3 h at an MOI of 1:8, while maintaining the same concentrations of indole derivatives as in the priming stage. Thereafter, cells were washed with PBS and fixed with 4% paraformaldehyde in PBS for 15 min at room temperature to preserve cellular structures and permeabilized with 0.2% (v/v) Triton X-100 for 5 min at room temperature. Blocking and antibody incubations were carried out using a blocking solution of PBS containing 0.1% Triton X-100 and 5% bovine serum albumin (BSA). Bacteria were labeled using a rabbit anti-*E. coli* primary antibody (Abcam,1:1500) followed by incubation with an Alexa Fluor 488 conjugated secondary antibody (Abcam,1:1,000). Actin filaments were stained using fluorescently labeled phalloidin (0.2 μg/mL, Sigma). Coverslips were mounted using DAPI-containing mounting medium (Bar-Naor) to stain nuclei. Samples were imaged using a *Zeiss* fluorescence microscope with a × 63 objective, and images were acquired and analyzed with ZEN software.

### Statistical analysis

All experiments were performed at least in three independent biological replicates. Where applicable, technical replicates were included within each biological replicate. For statistical analysis, the mean of the technical replicates was calculated for each biological replicate, and statistical tests were performed between the biological replicate means. Statistical analyses were performed using GraphPad Prism software. Bacterial growth data were analyzed using a mixed-effects model followed by Dunnett’s multiple comparisons test. qPCR data were analyzed using ordinary one-way analysis of variance (one-way ANOVA) followed by Dunnett’s multiple comparisons test.

## Results

### Specific indole derivatives suppress EPEC T3SS activity in a dose-dependent manner

We have previously reported that indole reduces EPEC T3SS activity (Gorelik et al., 2022). To determine whether this effect is specific to indole or extends to other indole derivatives, we selected seven indole-related metabolites produced through bacterial tryptophan catabolism (Figure 1) and evaluated their effects on EPEC T3SS activity. T3SS activity was assessed by examining the levels of three T3SS translocators (EspA, EspB, and EspD) in the culture supernatants, as these proteins are secreted via the T3SS and serve as reliable markers of secretion activity (Slater et al., 2018; Abe et al., 1998; Gershberg et al., 2021; Hartland et al., 2000). In parallel, we examined the levels of the Tir effector in the bacterial pellets. Since Tir is not secreted/translocated in the absence of host cell signals, its levels in bacterial pellets serve as an indicator of T3SS-associated protein expression (Ide et al., 2003).

**Figure 2:**
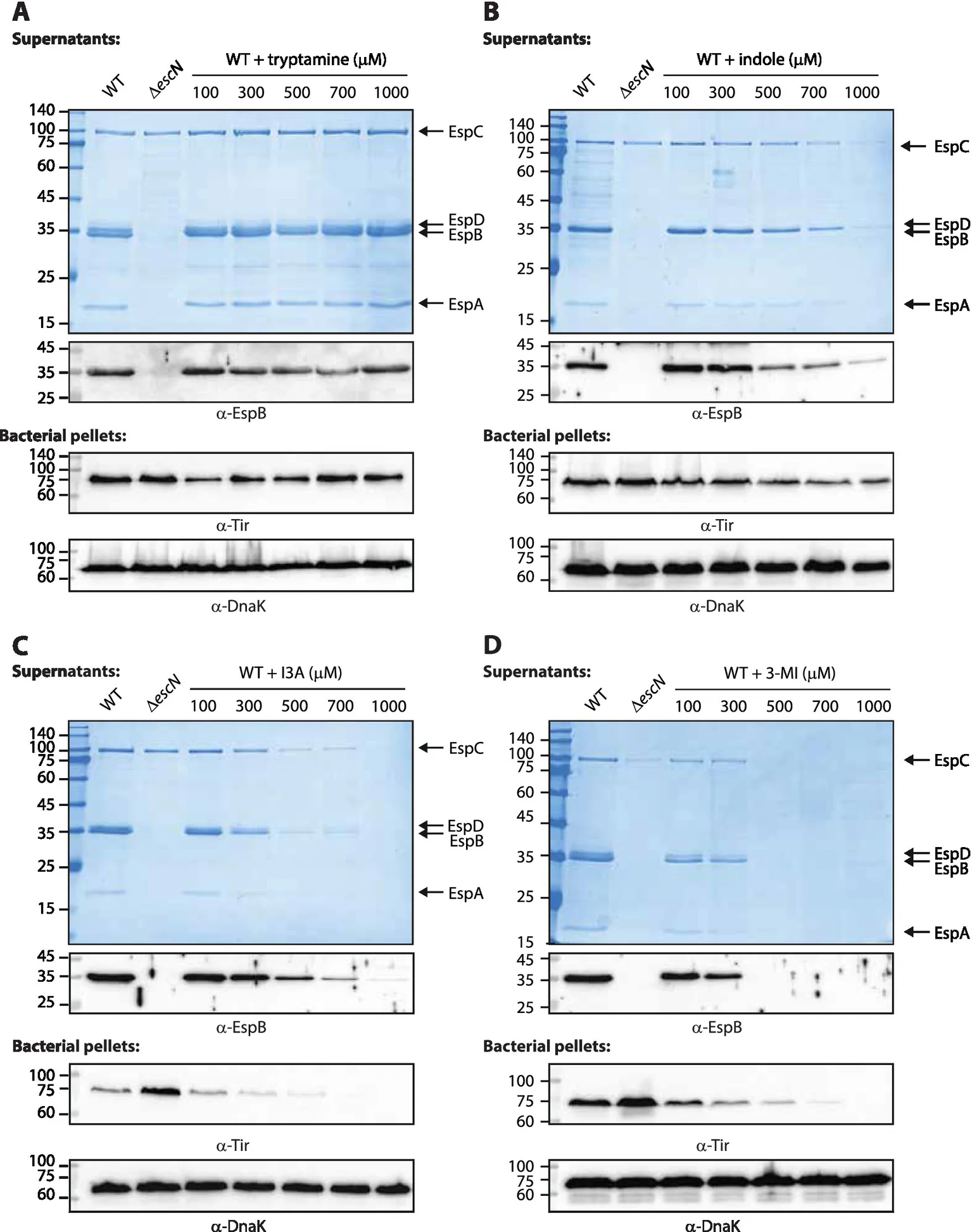
Specific indole derivatives inhibit EPEC T3SS activity in a dose-dependent manner. WT EPEC and Δ*escN* strains were grown under T3SS-inducing conditions either alone (1% DMSO vehicle control) or in the presence of increasing concentrations (100–1,000 μM) of tryptamine **(A)**, indole **(B)**, indole-3-carboxaldehyde (I3A, **C**), or 3-methylindole (3-MI, **D**). Culture supernatants were separated from bacterial pellets, normalized to bacterial density (OD_600_), and analyzed by SDS-PAGE followed by Coomassie Blue staining (top panels). The T3SS translocator proteins EspA, EspB, and EspD are indicated to the right of each gel, along with EspC, which is secreted independently of the T3SS. Supernatants and corresponding bacterial pellets were also analyzed by SDS-PAGE and western blotting using anti-EspB (supernatants) and anti-Tir (pellets) antibodies to detect the secreted translocator and intracellular effector, respectively. Anti-DnaK immunoblotting of pellet samples was used as a loading control. Each experiment was performed at least three times, and representative blots are shown.

WT EPEC and Δ*escN* cultures were grown under T3SS-inducing conditions (DMEM, CO_2_ incubator, statically) for 6 h. Following incubation, supernatant and pellet fractions were collected and analyzed by SDS-PAGE, followed by Coomassie staining or western blotting. As expected, we found that WT EPEC demonstrated T3SS activity, as reflected by the presence of EspA, EspB, and EspD in the supernatant sample (Coomassie staining, Figure 2). However, the *escN* mutant, which is deleted for the T3SS ATPase gene and therefore has a non-functional T3SS (Braverman et al., 2023), secreted no translocators (Coomassie staining, Figure 2). To assess the effect of indole derivatives on T3SS activity, WT EPEC cultures, grown under T3SS-inducing conditions, were supplemented with the compounds at concentrations ranging from 100 to 1,000 μM, and secretion level levels were evaluated. Of the seven indole derivatives examined, five exhibited no effect or only minor effects on EPEC T3SS activity. Tryptamine is shown in Figure 2A as a representative example of this group, while the results of the remaining derivatives - tryptophol, indole-3-acetic acid (IAA), indole-3-lactic acid (I3LA), and indole-3-propionic acid (I3PA) - are presented in Supplementary Figure S1. Tryptamine exhibited no detectable impact on T3SS activity; secretion of EspA, EspB, and EspD remained comparable across all tested concentrations, as shown by Coomassie blue staining (Figure 2A). Consistently, western blot analysis revealed no reduction in EspB secretion or Tir expression in the bacterial pellets, indicating that tryptamine does not alter T3SS-associated protein expression or secretion.

As expected, indole exhibited a gradual, dose-dependent reduction in secretion, with faint bands detectable at 1,000 μM (Figure 2B). The effect of indole on Tir expression was considerably milder. Incubation of WT EPEC with indole-3-carboxaldehyde (I3A) resulted in a stronger inhibition of T3SS activity compared with indole, as evidenced by a marked reduction in EspB secretion and Tir expression at 500 μM (Figure 2C). Among all derivatives tested, 3-methylindole (3-MI) showed the most pronounced effect, causing an almost complete loss of detectable secretion at concentrations of 500 μM and above (Figure 2D). DnaK levels remained consistent across all samples, confirming that the observed effects were not due to differences in bacterial loading. Overall, these results identified specific indole derivatives with strong inhibitory activity of EPEC T3SS.

**Figure 3:**
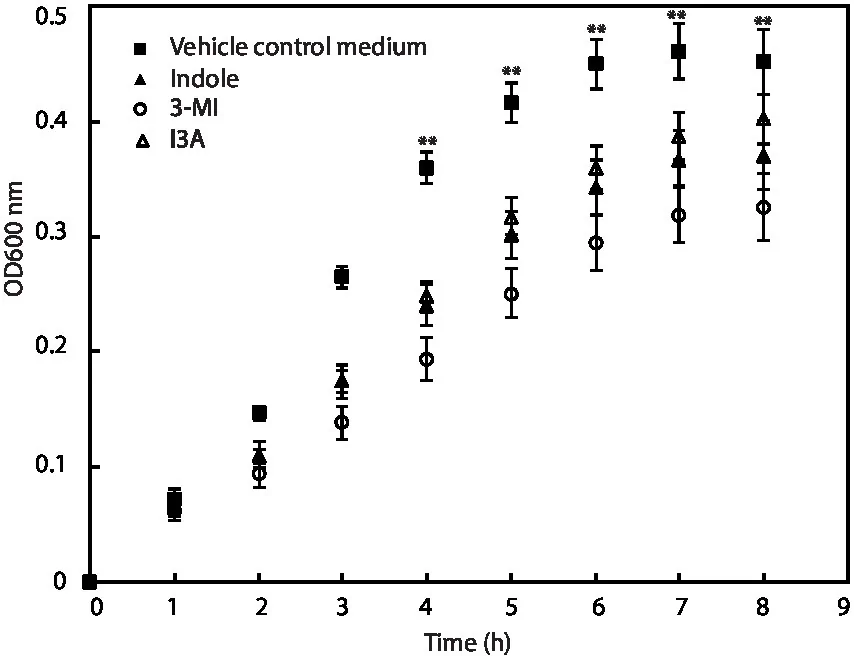
Effect of indole, 3-methylindole (3-MI), and indole-3-carboxaldehyde (I3A) on EPEC fitness. WT EPEC were cultured in phenol-free DMEM containing 1% DMSO (vehicle control) either alone or supplemented with indole, 3-MI, or I3A (1,000 μM). Bacterial growth was monitored over 8 h by measuring OD_600_. Each condition was tested in biological replicates and repeated at least three times. Bacteria grown in vehicle control medium are shown as black squares, in culture supplemented with indole as black triangles, in culture supplemented with 3-MI as open circles, and in culture supplemented with I3A as open triangles. Bacterial growth data were analyzed using a mixed-effects model followed by Dunnett’s multiple-comparisons test. Significant differences compared with the vehicle-treated WT EPEC were observed from 4 h onward (*p* < 0.01, **).

To assess whether the indole-derivatives induce a bacteriostatic/bactericidal effect, we monitored EPEC growth in the presence of the highest indole derivative concentration tested in the T3SS activity assay (1,000 μM). WT EPEC was cultured in DMEM under T3SS-inducing conditions in the presence of indole, 3-MI, or I3A, and bacterial growth was measured over an 8 h period by monitoring OD_600_ (Figure 3). We found that all three compounds led to a statistically significant reduction in growth compared to bacteria grown in DMEM medium with DMSO only from 4 h onward. Indole and I3A produced only a mild decrease in growth, whereas 3-MI had a more pronounced effect. Nevertheless, despite the observed growth rate reduction, the bacteria continued to grow throughout the experiment, thus suggesting that these compounds do not induce a bacteriostatic/bactericidal effect. Overall, because the T3SS assay samples were normalized to bacterial content, the growth reduction observed in the presence of indole derivatives cannot account for the observed suppression of T3SS activity.

### Indole derivatives inhibit bacterial infection and T3SS-mediated effector translocation *in vitro*

**Figure 4:**
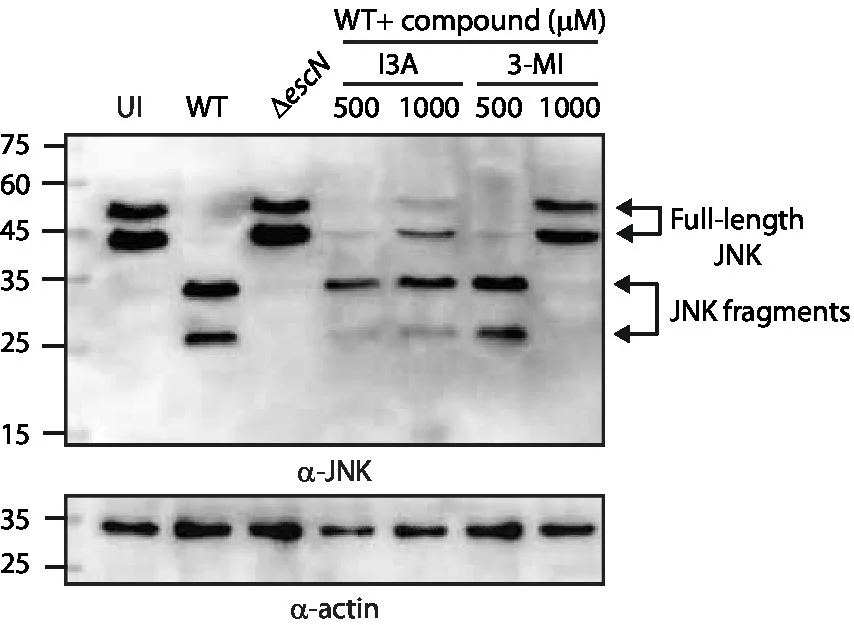
Indole derivatives reduce the ability of EPEC to infect cells *in vitro*. HeLa cells were infected with WT and Δ*escN* EPEC strains grown under T3SS-inducing conditions either alone (1% DMSO vehicle control) or in the presence of 500 μM or 1,000 μM 3-MI or I3A. Following infection, cells were lysed and analyzed by western blot using anti-JNK and anti-actin (loading control) antibodies. Full-length JNK (two isomers) and its degradation fragments are indicated at the right of the gel. Uninfected cells (UI) serve as a negative control. Each experiment was performed at least three times, and representative blots are presented.

To determine whether inhibition of T3SS activity with specific indole derivatives affects the ability of EPEC to deliver effector proteins into host cells, we measured the cleavage of cellular c-Jun N- terminal kinase (JNK), which is degraded by the NleD effector (Baruch et al., 2011). Because NleD-mediated degradation of JNK occurs only when NleD is successfully translocated, JNK cleavage serves as a functional indicator for T3SS-mediated effector delivery into host cells. HeLa cells were infected with WT EPEC or Δ*escN* strains alone or in the presence of indole derivatives, and JNK cleavage was analyzed by western blotting (Figure 4). As expected, uninfected cells and cells infected with the Δ*escN* mutant displayed the two full-length JNK isomers, with no detectable cleavage products. In contrast, infection with WT EPEC resulted in complete JNK degradation, as indicated by the disappearance of the bands corresponding to full-length JNK and the appearance of cleavage fragments (Figure 4). Infection of HeLa cells by WT EPEC in the presence of I3A showed a dose-dependent inhibition of JNK cleavage, with full-length JNK detected alongside its cleavage fragments (Figure 4). Even at 1,000 μM, inhibition remained partial, as indicated by stronger bands corresponding to the full-length JNK and a reduction, but not complete loss, of the cleavage fragments. Since complete inhibition was not achieved at this concentration, we tested a higher concentration (2,000 μM) to determine whether full suppression of effector translocation could be obtained. Cell infection with WT EPEC in the presence of 2,000 μM concentration of I3A resulted in complete inhibition of JNK cleavage (Supplementary Figure S2), suggesting that I3A can induce significant translocation inhibition when added at high concentration. To account for the slower bacterial growth observed in the presence of 2,000 μM I3A and indole (Supplementary Figure S3), a higher bacterial inoculum was used under these conditions.

Interestingly, incubation of WT EPEC with 3-MI produced a more pronounced inhibition of bacterial infection and effector translocation. At 500 μM, JNK degradation remained evident, but at 1,000 μM, JNK cleavage was completely abolished, as indicated by strong full-length JNK bands and the absence of detectable cleavage fragments (Figure 4). These findings indicate that the ability of indole derivatives to inhibit effector translocation into host cells is strongly dependent on both the specific compound and its concentration. To confirm that the reduction in JNK degradation by 3-MI and I3A were selectively driven by virulence attenuation rather than a general cellular stress response, we evaluated the host-cell cytotoxicity induced by the indole derivatives. For this purpose, HeLa cells were incubated with indole, 3-MI, or I3A at various concentrations for 4 h. Cytotoxicity was assessed by measuring LDH release into the culture medium. No significant increase in LDH release was observed in cells treated with indole, 3-MI, or I3A (Supplementary Figure S4). These results indicate that the observed inhibition of T3SS-dependent virulence is unlikely to result from host-cell cytotoxicity under the experimental conditions used in this study.

### 3-MI impairs EPEC-mediated actin pedestal formation

**Figure 5:**
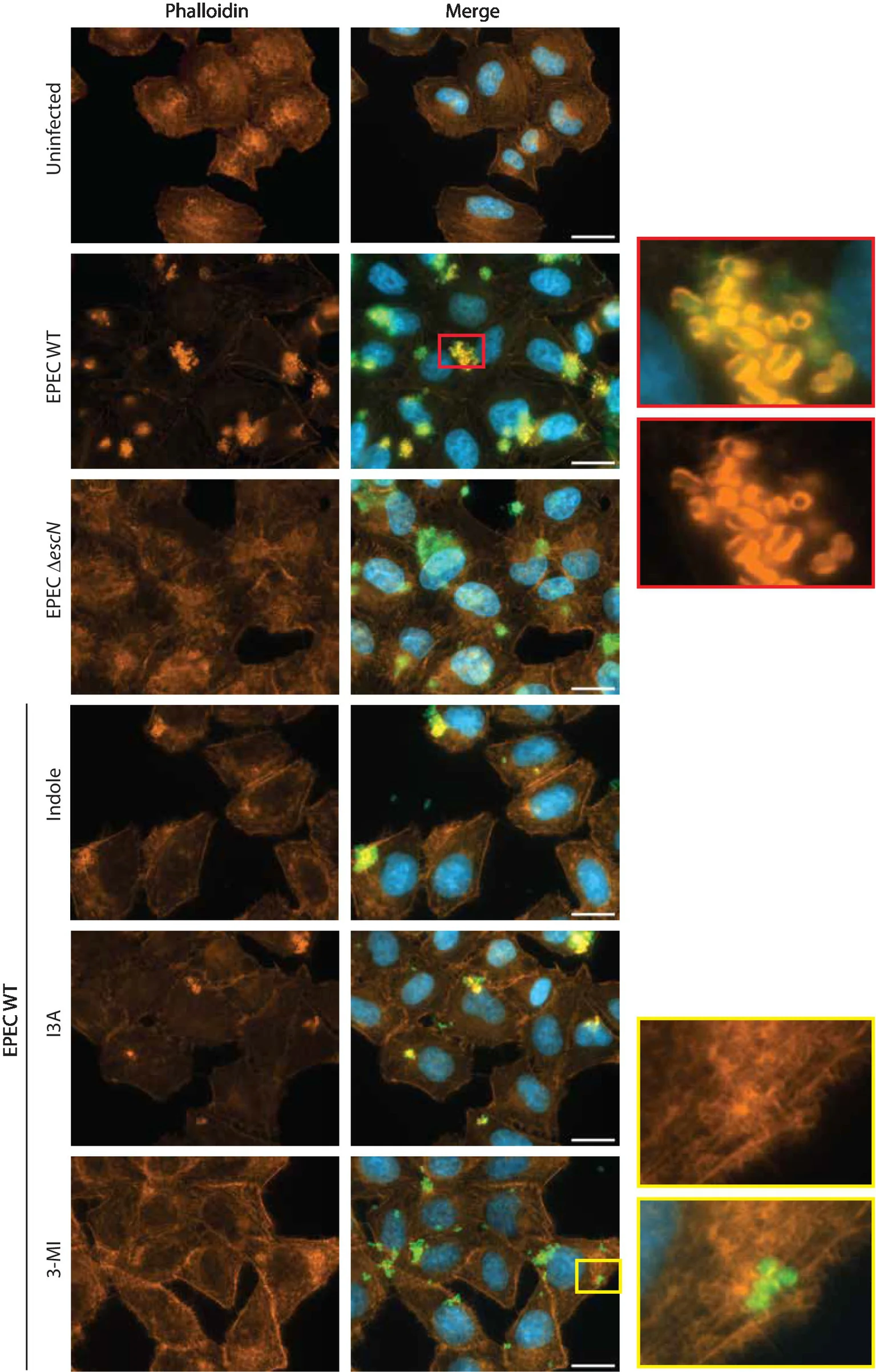
Indole derivatives impair EPEC-induced actin pedestal formation in host cells. HeLa cells were infected with WT EPEC either alone (2% DMSO vehicle control) or in the presence of indole (2,000 μM), 3-MI (1,000 μM), or I3A (1,000 μM). Cells infected with the Δ*escN* strain served as a negative control. Cells were then washed, fixed, and permeabilized. Actin filaments were stained with phalloidin (F-actin) and nuclei with DAPI (DNA). Bacteria were detected using a rabbit anti-*E. coli* antibody followed by an Alexa Fluor 488-conjugated secondary antibody. Higher-magnification phalloidin (bottom) and merged (top) images reveal that WT-infected cells (outlined in red) display clear actin pedestals, while 3-MI-treated samples (outlined in yellow) show no such structures. Scale bar: 20 μm. Images are representative of at least three independent experiments.

Actin pedestal formation is a hallmark of EPEC virulence and serves as a useful indicator of its ability to infect host cells (Battle et al., 2014; Dhanda et al., 2021; Goosney et al., 2001). To examine how indole derivatives affect this process, we infected HeLa cells with either WT EPEC or the Δ*escN* mutant in the absence or the presence of indole, I3A, or 3-MI. Following infection, cells were washed, fixed, and stained with phalloidin to visualize actin filaments, DAPI to visualize nuclei, and an anti-EPEC antibody to detect bacteria (Swistak et al., 2025; Vogt et al., 2023). Cells infected with WT EPEC exhibited robust bacterial attachment, with numerous actin pedestals beneath attached bacteria, whereas uninfected cells showed no detectable bacteria or pedestal structures (Figure 5). Infection of cells with the Δ*escN* mutant showed bacterial attachment but failed to form pedestals, confirming that a functional T3SS is required for pedestal formation. Pre-treatment of bacteria with indole (used at 2,000 μM due to its moderate effect on host infection; Supplementary Figure S2) and I3A (1,000 μM) reduced pedestal formation compared to WT EPEC infection, although pedestals remained detectable. Remarkably, 3-MI (1,000 μM) caused near-complete loss of pedestal formation despite bacterial attachment, indicating that it disrupts T3SS-mediated host cell interaction rather than bacterial binding.

### I3A and 3-MI suppress T3SS gene expression

**Figure 6:**
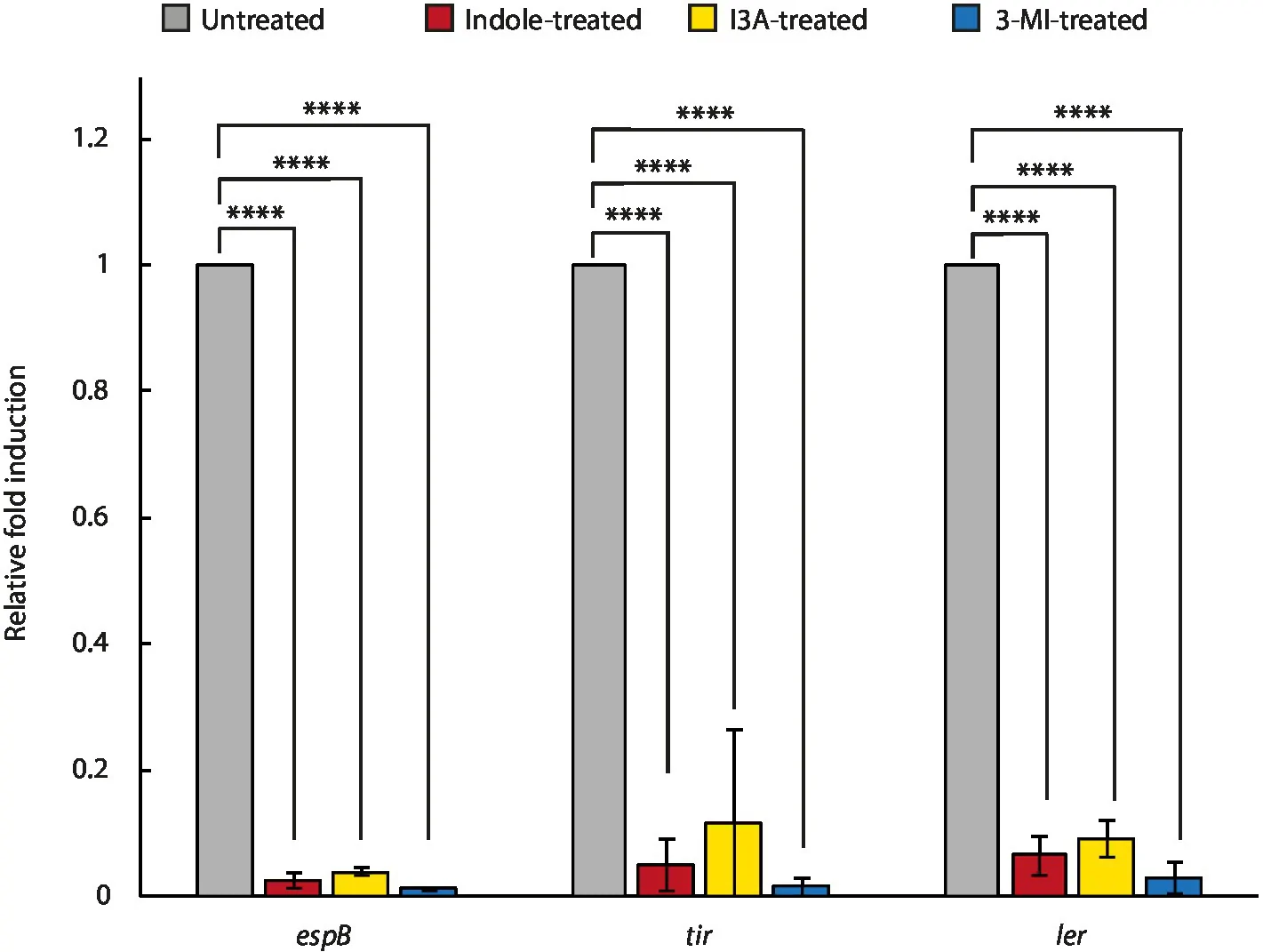
Indole derivatives modulate T3SS gene expression in EPEC. Quantitative real-time PCR (qPCR) was used to evaluate the effect of indole derivatives on the expression of T3SS-related virulence genes (*espB, tir,* and *ler*) of EPEC. WT EPEC cultures were grown under T3SS-inducing conditions and treated with indole (2,000 μM), 3-MI (1,000 μM), or I3A (1,000 μM). Total RNA was extracted, reverse transcribed into cDNA, and gene expression levels were quantified using gene-specific primers. Transcript levels were normalized to the housekeeping gene *rpoA* and are presented as relative expression compared to the vehicle control. Gray bars represent WT EPEC grown in DMEM alone (2% DMSO vehicle control); indole-treated samples are shown in red, I3A-treated samples in yellow, and 3-MI-treated samples in blue. Data represent the mean ± SEM of independent biological replicates. Statistical analysis was performed using one-way ANOVA. Significant differences compared with the vehicle-treated WT EPEC are indicated as (****, *p* < 0.0001).

To determine whether the reduction in T3SS activity observed upon addition of I3A or 3-MI is accompanied by changes in gene transcription, we extracted RNA from EPEC cultures grown in DMEM alone or with indole, I3A, or 3-MI. The RNA was reverse-transcribed into cDNA and used to assess the transcription levels of three representative T3SS genes: *ler*, *espB*, and *tir*. We found that bacteria exposed to indole, I3A, or 3-MI exhibited a significant decrease in the transcription of all three genes relative to untreated controls, with 3-MI producing the strongest inhibitory effect (Figure 6). These results indicate that indole derivatives suppress T3SS activity, at least in part, by downregulating the transcription of virulence-associated genes.

### I3A and 3-MI modulate the T3SS activity of additional enteric pathogens

To investigate whether the T3SS-inhibitory effect of indole derivatives is specific to EPEC or extends to additional human pathogens, we examined two additional foodborne pathogens that cause severe gastrointestinal illness: EHEC and *Salmonella enterica* serovar Typhimurium (*S.* Typhimurium).

**Figure 7:**
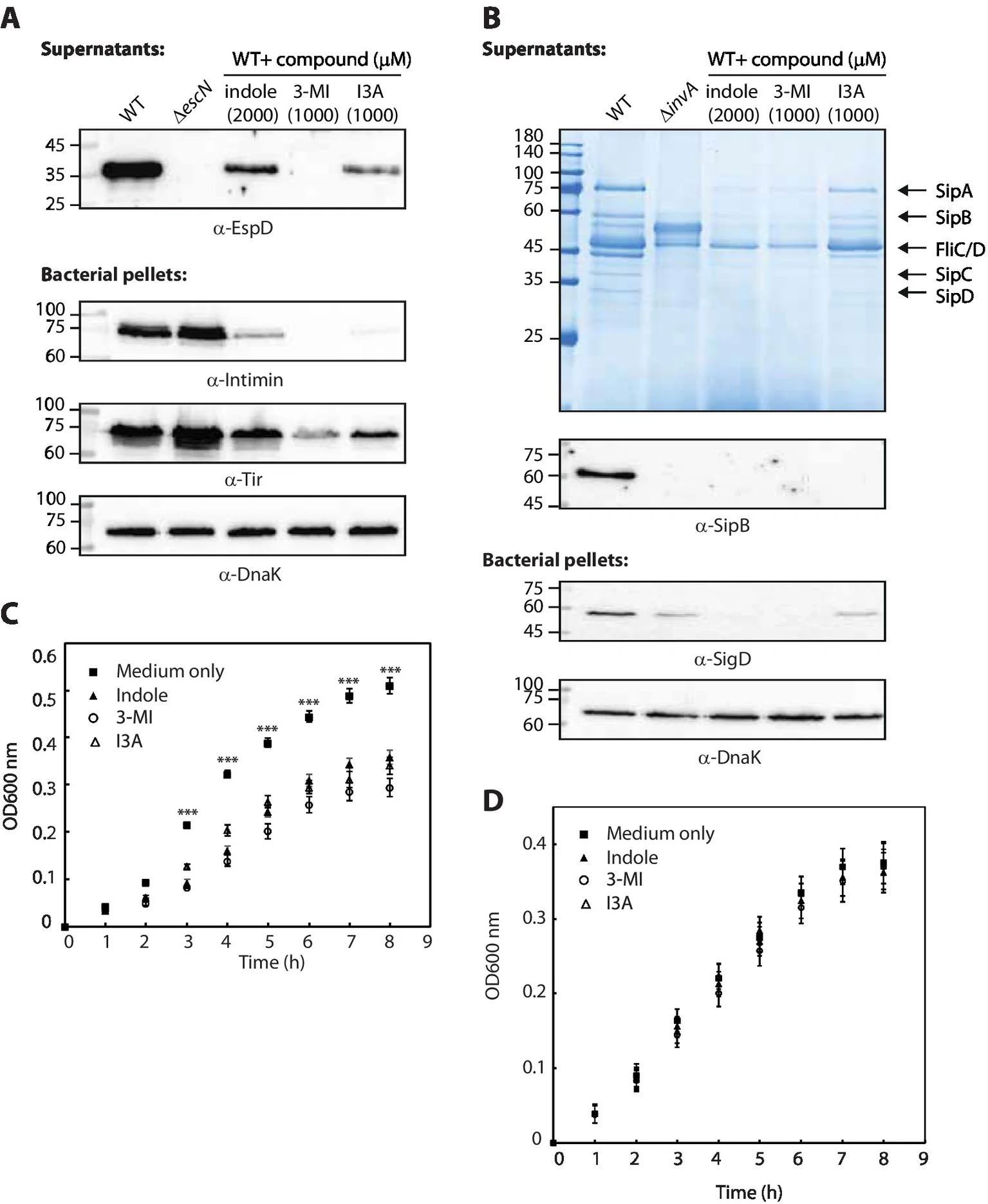
Indole derivatives suppress T3SS activity across multiple enteric pathogens. **(A)** EHEC WT and Δ*escN* strains were grown under T3SS-inducing conditions for 8 h in the presence or absence of indole (2,000 μM), 3-MI (1,000 μM), or I3A (1,000 μM). Culture supernatants and bacterial pellets were separated and analyzed by Western blot using antibodies against the secreted translocator EspD, as well as T3SS-associated proteins Tir and intimin. Anti-DnaK was used as a loading control. **(B)** *S.* Typhimurium WT and Δ*invA* strains were grown for 4 h in the presence or absence of indole, 3-MI, or I3A. Culture supernatants and bacterial pellets were separated and analyzed by Western blot using antibodies against the secreted translocator SipB and the T3SS-associated protein SigD, with DnaK serving as a loading control. In addition, secreted proteins were visualized by SDS-PAGE followed by Coomassie Blue staining, revealing the major T3SS substrates SipA, SipB, SipC, SipD, and flagellin (FliC/D). Each experiment was performed at least three times, and representative blots are presented. The growth of EHEC cultured in DMEM **(C)** and *Salmonella* cultured in LB **(D)**, with and without indole derivatives, was monitored over 8 h by measuring OD_600_. Bacteria grown in DMEM alone (2% DMSO vehicle control) are shown as black squares, indole-treated samples (2,000 μM) as black triangles, 3-MI-treated samples (1,000 μM) as open circles, and I3A-treated samples (1,000 μM) as open triangles. Bacterial growth data were analyzed using a mixed-effects model followed by Dunnett’s multiple-comparisons test. Significant differences compared with the vehicle-treated WT bacteria were observed for EHEC **(C)** from 3 h onward (***, *p* < 0.001), whereas no significant differences were observed for *Salmonella* **(D)**.

WT EHEC and Δ*escN* strains were grown under T3SS-inducing conditions alone or in the presence of indole, 3-MI, or I3A. Supernatant fractions were separated from the bacterial pellets and analyzed for T3SS activity. Because EHEC generally produces substantially lower levels of secreted proteins than EPEC, T3SS-dependent secretion was difficult to assess by Coomassie staining and was therefore evaluated by western blot analysis. As the EspB antibody used in our EPEC assays exhibits reduced sensitivity toward the EHEC EspB protein, we employed an antibody against the translocator protein EspD as a more reliable marker of T3SS activity in EHEC. EspD secretion was clearly detected in the supernatants of WT EHEC bacteria, whereas no secretion was observed in the supernatant of the EHEC Δ*escN* strain (Figure 7A). Incubation of WT EHEC with indole and its derivatives reduced EspD secretion, with the strongest inhibition observed in 3-MI-treated samples. The results from bacterial pellets were consistent with the secretion profiles observed in the supernatant samples; Tir and Intimin levels were high in both WT EHEC and the Δ*escN* mutant, but were reduced following incubation with indole, with an even more pronounced decrease in samples treated with 3-MI or I3A (Figure 7A). Notably, this trend was observed despite indole being tested at a higher concentration (2,000 μM) than for I3A and 3-MI (1,000 μM), further underscoring the stronger inhibitory effects of these indole derivatives.

To evaluate the T3SS activity in *S.* Typhimurium, we cultured WT and Δ*invA* strains in the presence or absence of indole derivatives. Because *invA* encodes an essential structural component of the T3SS apparatus, the Δ*invA* mutant serves as a T3SS-deficient negative control. *Salmonella* cultures were grown under T3SS-inducing conditions (LB broth, shaking), and supernatants were separated from bacterial pellets for analysis by SDS-PAGE and Coomassie staining. As expected, multiple T3SS-secreted proteins, including SipA, SipB, SipC, and SipD, were detected in the supernatant of WT *Salmonella* along with flagellar proteins, such as FliC and FliD (Cornelis, 2006; Komoriya et al., 1999), whereas only the flagellar proteins were detected in the Δ*invA* supernatant samples (Figure 7B, Coomassie). Western blot analysis further confirmed that SipB was secreted by WT bacteria but absent in the Δ*invA* strain. Incubation of WT *Salmonella* with indole derivatives resulted in reduced T3SS activity, as indicated by lower secretion in Coomassie-stained gels and reduced SipB detection in western blots (Figure 7B). The results from bacterial pellets analyzed by western blot with the anti-SigD antibody (detecting the SigD effector, which is retained intracellularly in the absence of host cells) were consistent with the secretion patterns observed in the supernatant samples. SigD levels were high in WT bacteria but reduced in both the Δ*invA* mutant and the indole derivatives-treated samples, while DnaK levels remained unchanged (Figure 7B).

To assess whether the indole-derivatives exert a bacteriostatic or bactericidal effect on EHEC or *Salmonella*, we monitored the bacterial growth under T3SS-inducing conditions in the presence of indole, 3-MI, and I3A. In EHEC, all indole derivatives caused a slight to moderate reduction in growth, with 3-MI exerting the strongest effect (Figure 7C). In contrast, *Salmonella* showed no measurable growth defect in the presence of indole derivatives (Figure 7D). Although indole derivatives led to partial inhibition of EHEC growth, this alone cannot explain the inhibition of T3SS activity, as all samples were normalized to bacterial content, ensuring that reductions in secretion reflect true impairment of T3SS activity. Overall, these findings indicate that 3-MI and I3A reduce T3SS activity across multiple enteric pathogens.

## Discussion

Our study finds that indole derivatives, naturally produced by gut bacteria, attenuate T3SS-dependent virulence in several enteric pathogens. While indole itself exerted moderate inhibition (Franco et al., 2025; Gorelik et al., 2022; Janiga-MacNelly et al., 2025), its derivatives, 3-MI and I3A, consistently demonstrated stronger effects across assays. The fact that only a subset of structurally related metabolites exhibited activity highlights a clear structure–function relationship, suggesting that specific chemical features of the indole scaffold are required for anti-virulence activity (Ye et al., 2022; Powell et al., 2020; Janiga-MacNelly et al., 2025; Sethupathy et al., 2020).

Comparison of the examined metabolites suggests that the structural organization and physicochemical properties of the substituent at the 3-position of the indole ring are critical determinants of activity. The three most active compounds contain relatively small, rigid, and compact substituents that preserve the planar indole scaffold. Notably, the two most active derivatives, 3-MI and I3A, contain substituents directly linked to the indole ring without an intervening methylene spacer, whereas less active metabolites generally contain one or more methylene groups separating the indole core from their terminal functional groups. These structural differences may influence bacterial uptake or binding to intracellular targets.

Our findings suggest that 3-MI and I3A act, at least in part, at the transcriptomic level by reducing the expression of key LEE-encoded genes, including *ler*, *espB*, and *tir*, ultimately diminishing secretion and translocation of T3SS substrates. The alignment of transcriptional repression with downstream functional outcomes supports a model in which these metabolites impact upstream regulatory nodes that coordinate virulence activation. This observation is significant, as it suggests that bacteria exposed to 3-MI and I3A can remain physiologically viable while their pathogenic capabilities are selectively attenuated. This distinction is critical and aligns with a growing trend in antimicrobial research to prioritize anti-virulence strategies over bactericidal approaches (Dickey et al., 2017; Fleitas Martinez et al., 2019; Ivanov et al., 2022). Identifying metabolites that exert anti-virulence effects without killing bacteria could offer a novel therapeutic avenue for combating infectious diseases. Unlike conventional antibiotics, such an approach would minimize selective pressure for resistance development, potentially extending the clinical utility of antimicrobial interventions.

A key aspect of this study is that the observed anti-virulence activity is not restricted to EPEC but extends to other enteric pathogens, including EHEC and *S. enterica*, despite differences in their T3SS architecture and regulation. This suggests that indole-derived metabolites may target conserved regulatory circuits governing T3SS expression across diverse pathogenic species. Beyond the T3SS, 3-MI and I3A have been reported to modulate other virulence-associated phenotypes, including biofilm formation and quorum-sensing-linked regulation in various bacterial systems (Bommarius et al., 2013; Choi et al., 2014; Odularu et al., 2022). Together, these findings indicate that the inhibitory activity of these metabolites reflects a broader capacity to modulate bacterial pathogenesis. Because T3SS expression is highly responsive to environmental cues, the magnitude and nature of the effects exerted by indole derivatives may differ under alternative environmental conditions. Accordingly, our findings should be interpreted in the context of the T3SS-inducing conditions used in this study. Future studies are needed to determine whether these effects are consistent under physiological gut conditions.

An important factor in evaluating the therapeutic potential of these molecules is the concentration at which they exert biological effects while remaining safe. While certain indole derivatives are reported to exhibit cytotoxicity in mammalian systems when added at high concentrations, I3A is distinct in its well-characterized role as a gut-health-promoting metabolite that strengthens intestinal barrier integrity via AhR activation (D'Onofrio et al., 2021; Lu et al., 2023; Xie et al., 2024). In addition, although the concentration of indole within the intestinal lumen has not been precisely established, previous studies measuring fecal indole levels in healthy adults suggest that millimolar concentrations are physiologically relevant (Darkoh et al., 2015; Karlin et al., 1985). Darkoh et al. (2015) reported mean and median fecal indole concentrations of 2.59 and 2.73 mM, respectively, with values ranging from 0.3 to 6.64 mM across individuals. Notably, the highest frequency of samples contained indole concentration within the 2–3 mM range. Moreover, because indole is readily absorbed across the colonic epithelium, luminal indole concentrations may be higher than those measured in fecal samples (Tennoune et al., 2022). Collectively, these observations suggest that the 1–2 mM indole derivative concentrations used in the present study are within a physiologically relevant range for exposure in the human intestine.

Given these considerations, the concentrations of I3A used in our study fall within a biologically active range that inhibits T3SS without compromising host-cell viability. Consistent with this, LDH release assays performed under the experimental conditions used in the present study did not reveal a statistically significant increase in LDH release following treatment with either I3A or 3-MI compared with the corresponding vehicle control. However, previous studies have reported that 3-MI can exhibit cytotoxic effects in mammalian cells at relatively low to moderate micromolar concentrations (Nichols et al., 2003; Updyke et al., 1991). Taken together, these findings suggest that the observed anti-virulence activity is not associated with significant host-cell cytotoxicity under the conditions tested in this study, although potential cytotoxic effects under different cell types, exposure times, concentrations, or experimental settings cannot be excluded. Therefore, 3-MI represents a promising anti-virulence candidate, albeit one that warrants caution in therapeutic development in light of previous cytotoxicity reports (Janiga-MacNelly et al., 2025; Kumar et al., 2021; Smits et al., 2024). A combinatorial approach using 3-MI together with I3A may permit the use of lower concentrations of 3-MI while preserving anti-virulence activity and potentially improving the safety profile of future therapeutic applications. This will better reflect the intestinal environment, where different indole metabolites coexist as part of a complex mixture of microbiome-derived compounds rather than acting individually. In the present study, each metabolite was intentionally evaluated independently to determine its intrinsic anti-virulence activity and to enable direct comparison of their relative effects under identical experimental conditions. Although this reductionist approach allowed identification of the most active metabolites, it does not account for potential additive, synergistic, or antagonistic interactions that may occur *in vivo*. Additional research investigating combinations of microbiome-derived indole metabolites will therefore be important to determine how these compounds collectively influence T3SS regulation and bacterial virulence under physiologically relevant conditions.

Among the compounds tested, 3-MI emerged as the most potent inhibitor across all T3SS-related assays, including transcription, secretion, effector translocation, and pedestal formation. This consistent phenotype highlights its potential as a lead compound for anti-virulence strategies. More broadly, our findings support the concept that targeting virulence rather than bacterial growth represents a promising approach to mitigating infection. Future studies should explore the potential of 3-MI and I3A as postbiotics, functional bioactive metabolites produced by the gut microbiome that confer health benefits to the host. Such interventions could enhance colonization resistance to infectious diseases in susceptible individuals and support recovery following enteric infections.

## Data Availability

The raw data supporting the conclusions of this article will be made available by the authors, without undue reservation.
