# Supplementary material for: Gut microbiome-derived indoles differentially suppress type III secretion system-associated virulence in Escherichia coli and Salmonella enterica
Source: Front Microbiol. 2026 Aug 13;17:1902638. doi: 10.3389/fmicb.2026.1902638 (PMC13518527; doi:10.3389/fmicb.2026.1902638)
Supplement: Supplementary file 1 [file Data_Sheet_1.DOCX]

**Figure S1. Indole derivatives with no significant effect on EPEC T3SS activity.** WT EPEC and Δ*escN* strains were grown under T3SS-inducing conditions either alone or in the presence of increasing concentrations (100-1000 μM) of tryptophol (A), indole-3-acetic acid (IAA, B), indole-3-lactic acid (I3LA, C), or indole-3-propionic acid (I3PA, D). Culture supernatants were separated from bacterial pellets, normalized to bacterial density (OD_600_), and analyzed by SDS-PAGE followed by Coomassie Blue staining (top panels). The T3SS translocator proteins EspA, EspB, and EspD are indicated to the right of each gel, along with the position of EspC, which is secreted independently of the T3SS. Supernatants and corresponding bacterial pellets were also analyzed by SDS-PAGE and western blotting using anti-EspB (supernatants) and anti-Tir (pellets) antibodies to detect the secreted translocator and intracellular effector, respectively. Anti-DnaK immunoblotting of pellet samples was used as a loading control. Each experiment was performed at least three times, and representative blots are presented. WT cultures treated with DMSO alone (1% final concentration) served as the vehicle control.

**Figure S2. Indole and I3A impair NleD-mediated JNK degradation in a dose-dependent manner.** HeLa cells were infected with WT EPEC and Δ*escN* strains grown under T3SS-inducing conditions in the presence of increasing concentrations (500, 1000, or 2000 μM) of indole or I3A for 3 h. Following infection, host cells were lysed and analyzed by western blot using anti-JNK and anti-Actin (loading control) antibodies to assess effector-mediated JNK cleavage, with anti-actin serving as a loading control. Full-length JNK (two isomers) and its degradation fragments are indicated at the right of the gel. Uninfected cells (UI) serve as a negative control. Each experiment was performed at least three times, and representative blots are presented. WT cultures treated with DMSO alone (2% final concentration) served as the vehicle control.

**Figure S3. High concentrations of indole and I3A affect EPEC growth dynamics.** WT EPEC were cultured in DMEM containg DMSO (vehicle control) or supplemented with indole or I3A at a concentration of 2000 μM. Bacterial growth was monitored over 8 h by measuring OD_600_. Data represent biological replicates. Bacterial were grown in vehicle control medium are shown as black squares, indole-treated samples as black triangles, and I3A-treated samples as white triangles. Bacterial growth data were analyzed using a mixed-effects model followed by Dunnett's multiple-comparisons test. Significant differences compared with the vehicle-treated WT EPEC were observed from 3 h onward (***, P < 0.001).

**Figure S4. Active indole derivatives do not induce host-cell cytotoxicity.** HeLa cells were incubated with indole, 3-MI, or I3A at the same concentrations used in the JNK assay for 4 h. Cytotoxicity was assessed by measuring LDH release into the culture medium and normalizing values to the maximum-LDH-release control. No significant increase in LDH release was observed in cells treated with indole, 3-MI, or I3A compared with the corresponding vehicle control (DMSO at matched concentrations). Bars represent geometric means from quintuplicate measurements across three independent experiments. Error bars indicate standard deviation. Black bars represent the maximum LDH release control (LDH Max), dark gray bars represent untreated cells (cells only), light gray bars represent the corresponding DMSO vehicle controls; indole-treated samples are shown in red, I3A-treated samples in yellow, and 3-MI-treated samples in blue.
